# Global update on the susceptibility of human influenza viruses to neuraminidase inhibitors, 2015–2016

**DOI:** 10.1016/j.antiviral.2017.08.004

**Published:** 2017-10

**Authors:** Larisa V. Gubareva, Terry G. Besselaar, Rod S. Daniels, Alicia Fry, Vicki Gregory, Weijuan Huang, Aeron C. Hurt, Patricia A. Jorquera, Angie Lackenby, Sook-Kwan Leang, Janice Lo, Dmitriy Pereyaslov, Helena Rebelo-de-Andrade, Marilda M. Siqueira, Emi Takashita, Takato Odagiri, Dayan Wang, Wenqing Zhang, Adam Meijer

**Affiliations:** a WHO Collaborating Center for Surveillance, Epidemiology and Control of Influenza, Centers for Disease Control and Prevention (CDC), 1600 Clifton RD NE, MS-G16, Atlanta, GA, 30329, United States; b Global Influenza Programme, World Health Organization, Avenue Appia 20, 1211 Geneva 27, Switzerland; c The Francis Crick Institute, Worldwide Influenza Centre (WIC), WHO Collaborating Centre for Reference and Research on Influenza, 1 Midland Road, London, NW1 1AT, United Kingdom; d WHO Collaborating Centre for Reference and Research on Influenza, National Institute for Viral Disease Control and Prevention, Collaboration Innovation Centre for Diagnosis and Treatment of Infectious Diseases, China CDC, Beijing, China; e WHO Collaborating Centre for Reference and Research on Influenza, At the Peter Doherty Institute for Infection and Immunity, Melbourne, Victoria, 3000, Australia; f Department of Microbiology and Immunology, University of Melbourne, Parkville, Victoria, 3010, Australia; g National Infection Service, Public Health England, London, NW9 5HT, United Kingdom; h Public Health Laboratory Centre, 382 Nam Cheong Street, Hong Kong, China; i Division of Health Emergencies and Communicable Diseases, World Health Organization Regional Office for Europe, UN City, Marmorvej 51, DK-2100, Copenhagen, Denmark; j Influenza Pathogenesis and Antiviral Resistance Laboratory, National Institute of Health, Av. Padre Cruz, 1649-016, Lisboa, Portugal; k Faculdade de Farmácia, Universidade de Lisboa, Av. Prof Gama Pinto, 1649-016, Lisboa, Portugal; l National Influenza Center, Laboratorio de Virus Respiratorios, Oswaldo Cruz Institute/FIOCRUZ, Rio de Janeiro, Brazil; m WHO Collaborating Centre for Reference and Research on Influenza, National Institute of Infectious Diseases, Gakuen 4-7-1, Musashimurayama, Tokyo, 208-0011, Japan; n National Institute for Public Health and the Environment, PO Box 1, 3720 BA, Bilthoven, The Netherlands

**Keywords:** Neuraminidase, Inhibitor, Susceptibility, Surveillance, Resistance, Markers

## Abstract

Four World Health Organization (WHO) Collaborating Centres for Reference and Research on Influenza and one WHO Collaborating Centre for the Surveillance, Epidemiology and Control of Influenza (WHO CCs) assessed antiviral susceptibility of 14,330 influenza A and B viruses collected by WHO-recognized National Influenza Centres (NICs) between May 2015 and May 2016. Neuraminidase (NA) inhibition assay was used to determine 50% inhibitory concentration (IC_50_) data for NA inhibitors (NAIs) oseltamivir, zanamivir, peramivir and laninamivir. Furthermore, NA sequences from 13,484 influenza viruses were retrieved from public sequence databases and screened for amino acid substitutions (AAS) associated with reduced inhibition (RI) or highly reduced inhibition (HRI) by NAIs. Of the viruses tested by WHO CCs 93% were from three WHO regions: Western Pacific, the Americas and Europe. Approximately 0.8% (n = 113) exhibited either RI or HRI by at least one of four NAIs.

As in previous seasons, the most common NA AAS was H275Y in A(H1N1)pdm09 viruses, which confers HRI by oseltamivir and peramivir. Two A(H1N1)pdm09 viruses carried a rare NA AAS, S247R, shown in this study to confer RI/HRI by the four NAIs. The overall frequency of A(H1N1)pdm09 viruses containing NA AAS associated with RI/HRI was approximately 1.8% (125/6915), which is slightly higher than in the previous 2014-15 season (0.5%). Three B/Victoria-lineage viruses contained a new AAS, NA H134N, which conferred HRI by zanamivir and laninamivir, and borderline HRI by peramivir. A single B/Victoria-lineage virus harboured NA G104E, which was associated with HRI by all four NAIs. The overall frequency of RI/HRI phenotype among type B viruses was approximately 0.6% (43/7677), which is lower than that in the previous season.

Overall, the vast majority (>99%) of the viruses tested by WHO CCs were susceptible to all four NAIs, showing normal inhibition (NI). Hence, NAIs remain the recommended antivirals for treatment of influenza virus infections. Nevertheless, our data indicate that it is prudent to continue drug susceptibility monitoring using both NAI assay and sequence analysis.

## Highlights

• A total of 14,330 influenza viruses were collected worldwide, May 2015–May 2016.
• Approximately 0.8% showed reduced inhibition by at least one NA inhibitor.
• The frequency of viruses with reduced inhibition was slightly higher than in 2014–15 (0.5%).
• NA inhibitors remain an appropriate choice for influenza treatment.
• Global surveillance of influenza antiviral susceptibility should be continued.

## 1 Introduction

The M2 channel blockers are no longer recommended for treating seasonal influenza due to prevalent resistance (Bright et al., 2005, Nelson et al., 2009). The neuraminidase (NA) inhibitors (NAIs), which include oral oseltamivir, inhaled zanamivir, intravenous peramivir and inhaled long-acting laninamivir, have been used for controlling influenza virus infections. These inhibitors bind tightly to the active sites of influenza NAs, interfering with the enzymatic function required for efficient replication of the viruses and their mobility within an infected host (McKimm-Breschkin, 2013). Their availability and usage differ markedly, with oseltamivir being the most commonly prescribed in a number of countries, and laninamivir being licensed only in Japan (Ison, 2017, Kubo et al., 2010). In August 2016, the U.S. Food and Drug Administration (FDA) approved the first generic version of oseltamivir (FDA, 2016). There are substantial efforts to enhance the arsenal of anti-influenza drugs by developing compounds with alternative mechanisms of action (Naesens et al., 2016); in the meantime, NAIs remain the principal therapeutics to treat seasonal and zoonotic influenza virus infections.

WHO Global Influenza Surveillance and Response System (GISRS) laboratories around the world participate in surveillance efforts aimed at rapidly detecting the emergence of influenza strains with reduced susceptibility to NAI(s). The NAI assay is the primary tool utilized to monitor influenza susceptibility to NAIs through determination of the concentration of drug required to inhibit NA enzymatic activity by 50% (IC_50_). A threshold (cutoff IC_50_ value) which could be used to reliably separate drug-resistant and drug-sensitive viruses has not yet been established. Nevertheless, an elevated IC_50_ is generally viewed as an indicator of reduced antiviral activity of the NAI. To ensure consistency in interpreting and reporting of NAI assay data, the World Health Organization Expert Working Group on Surveillance of Influenza Antiviral Susceptibility (WHO-AVWG) introduced a set of criteria to define the antiviral susceptibility of viruses based on the fold change of their IC_50_. To this end, the IC_50_ of a test virus is compared to a reference IC_50_ (e.g. a median IC_50_ of viruses of the same type/subtype) to calculate a fold increase (WHO, 2012). Notably, the difference in the baseline IC_50_ values of influenza A and B viruses requires a type-specific interpretation of NAI assay data. Hence, for type A viruses, normal inhibition (NI) is reported when increases in IC_50_ of less than 10-fold are detected, reduced inhibition (RI) when 10- to 100-fold increases are observed and highly reduced inhibition (HRI) when increases of more than 100-fold are detected; for type B viruses the corresponding fold increases are <5, 5–50 and >50 respectively (WHO, 2012). Viruses displaying RI or HRI typically harbour changes (i.e. amino acid substitutions/deletions) within or near the NA active site which adversely affect binding of one or more NAIs (McKimm-Breschkin, 2013).

In 2007–2008, the unprecedented rise of seasonal A(H1N1) viruses carrying the NA amino acid substitution (AAS) H275Y was detected in Europe (Kawai et al., 2009); these oseltamivir-resistant viruses rapidly spread globally. The emergence of the pandemic A(H1N1) virus in 2009 ended their circulation, as they were displaced by the antigenically novel and oseltamivir-susceptible A(H1N1)pdm09 viruses (Garten et al., 2009). In subsequent seasons, A(H1N1)pdm09 viruses containing H275Y NA AAS have been detected in patients with and without exposure to oseltamivir treatment, with large clusters reported in Australia and Japan (Hurt et al., 2016, Meijer et al., 2014, Takashita et al., 2015a, Takashita et al., 2015b). The global spread of A(H1N1)pdm09 viruses carrying NA H275Y AAS remains the prime concern for public health because of evidence supporting their transmissibility in communities and dual resistance to oseltamivir and peramivir (Kelso and Hurt, 2012). Other less common NA AASs have also been reported to confer RI/HRI by NAI(s). Such AASs can occur in influenza A and B viruses, either spontaneously, or as a result of antiviral treatment. Hence, in order to develop strategies for pandemic preparedness and ensure appropriate treatment and clinical management guidelines, it is essential to monitor susceptibility to individual NAIs of all influenza types, subtypes and lineages.

## 2 Overall analysis of phenotypic antiviral susceptibility data from WHO CCs

The GISRS monitors the evolution of influenza viruses and provides recommendations on many influenza-related topics (http://www.who.int/influenza/gisrs_laboratory/en/). GISRS comprises 143 National Influenza Centres (NICs), 6 WHO Collaborating Centres (CCs), 4 WHO Essential Regulatory Laboratories, 13 WHO H5 reference laboratories and *ad hoc* groups established to address specific emerging issues. NICs collect virus specimens in their country and perform initial analysis. Representative viruses of each antigenic type and subtype/lineage are then shipped to one of the WHO CCs for further characterization. Virus specimens are commonly propagated in MDCK or MDCK-SIAT1 cells by WHO CCs prior to drug susceptibility assessment using the NAI assay (Hurt et al., 2012, World Health Organization, 2011). Viruses exhibiting RI or HRI are subjected to sequence analysis (together with their original clinical specimens if possible) to identify NA AASs responsible for the altered phenotype.

**Fig. 1:**
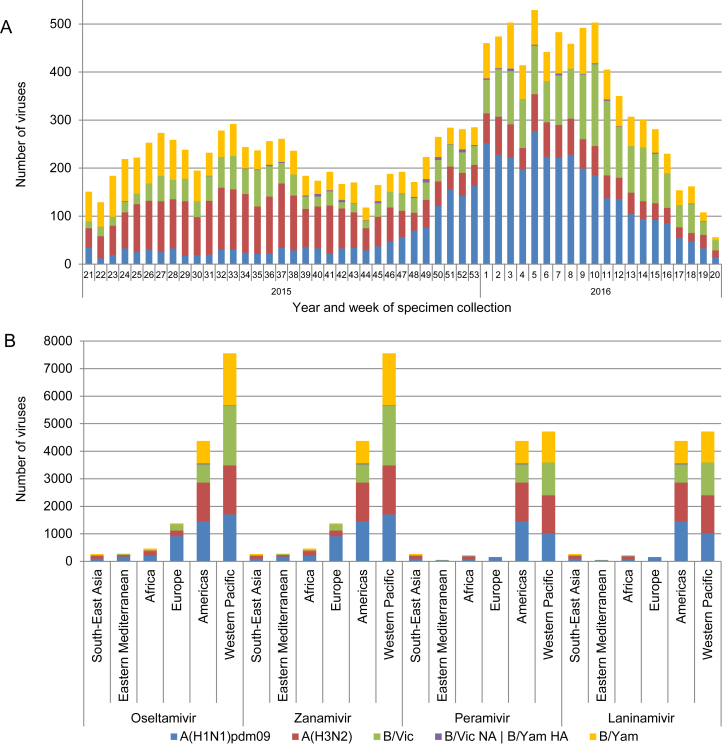
Influenza viruses collected and tested for phenotypic neuraminidase inhibitor (NAI) susceptibility during 2015–2016. A) Week of specimen collection and virus type/subtype/lineage; for specimens tested, peaks in specimen collection during the Southern Hemisphere winter and during the Northern Hemisphere winter were observed. B) Number of viruses tested for phenotypic susceptibility to the four NAIs by World Health Organization region. B/Yamagata-lineage haemagglutinin:B/Victoria-lineage neuraminidase reassortants are shown separately.

**Fig. 2:**
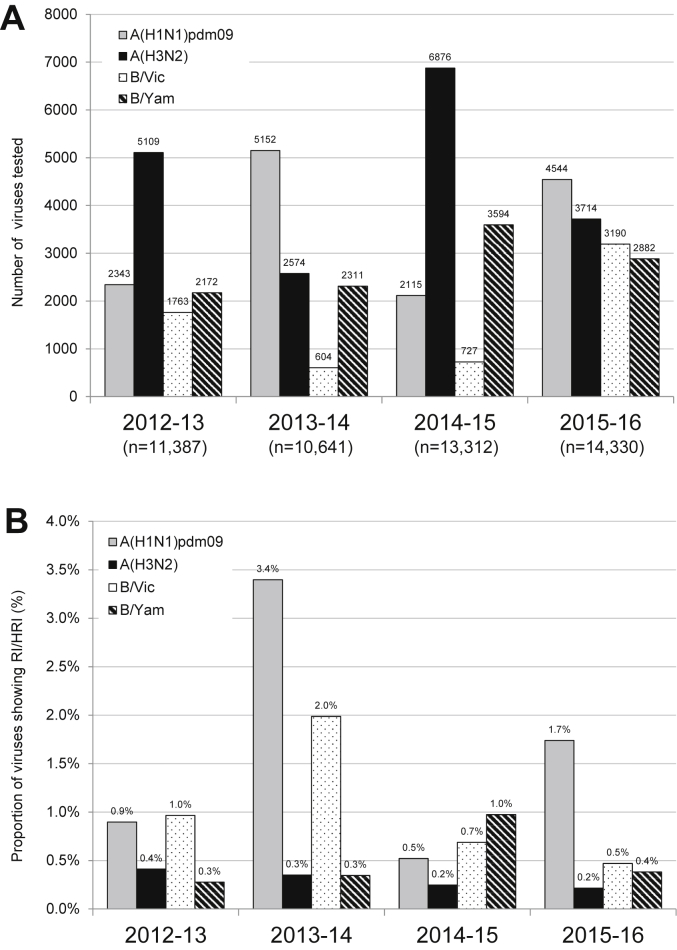
A) Number of viruses tested in the neuraminidase inhibition assays (NAI assay) over the 2012–2016 period. B) Proportion of viruses showing RI or HRI by neuraminidase inhibitors (NAIs) over the 2012–2016 period. Data compiled from the global studies reporting on viruses isolated during 2012–13 (Meijer et al., 2014), 2013–14 (Takashita et al., 2015b), 2014–15 (Hurt et al., 2016), and 2015–16 (current study). B/Yamagata-lineage haemagglutinin:B/Victoria-lineage neuraminidase reassortants are included in the proportion and number of B/Victoria-lineage viruses.

The data presented in this study includes the analysis of viruses collected between week 21/2015 (May 18, 2015) and week 20/2016 (May 22, 2016) (Fig. 1A). A total of 14,330 influenza viruses were phenotypically tested for susceptibility to oseltamivir and zanamivir (Fig. 1B and Fig. S1). Two-thirds of these viruses (n = 9795) were also tested for susceptibility to peramivir and laninamivir by the WHO CCs located in Atlanta, Melbourne and Tokyo (Fig. 1B). Compared to previous influenza seasons, the overall number of viruses tested increased by 7% (Fig. 2B). Among the viruses tested during 2015–16, A(H1N1)pdm09 viruses were most prevalent (4544; 31.7%), followed by A(H3N2) (3714; 25.9%), B/Victoria-lineage (3190; 22.3%) and B/Yamagata-lineage viruses (2882; 20.1%) (Fig. 2A).

Similar to previous global updates, the majority of viruses were submitted from the Western Pacific WHO region (52.8%), followed by the Americas (30.5%) and Europe (9.6%). Small proportions of the viruses were received from the WHO regions of Africa (3.2%), Eastern Mediterranean (2.0%) and South-East Asia (1.9%) (Fig. 1B).

**Fig. 3:**
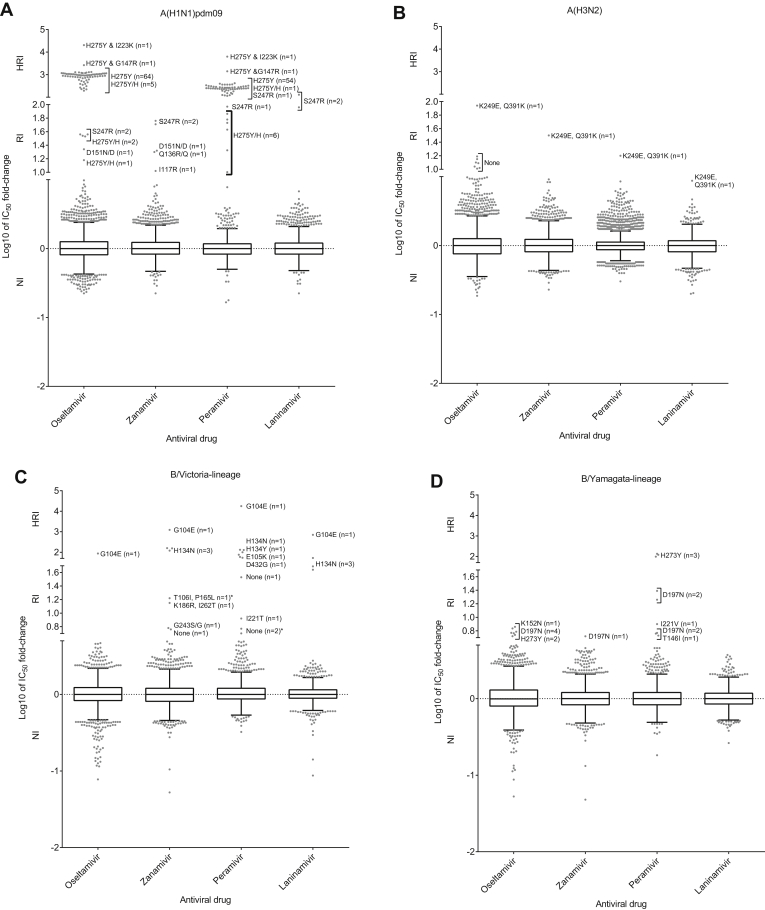
Column-scatter plots of log-transformed 50% inhibitory concentration (IC_50_) fold-change values. Data are presented by virus subtype or lineage [A) A(H1N1)pdm09; B) A(H3N2); C) B/Victoria-lineage; and, D) B/Yamagata-lineage] and neuraminidase inhibitor (labelled on the X-axis: oseltamivir, zanamivir, peramivir, laninamivir). Panel C) also contains B/Yamagata-lineage haemagglutinin:B/Victoria-lineage neuraminidase reassortants, of which the three with reduced inhibition are indicated with an asterix (*). The boxes indicate the 25–75 percentile and the whiskers stretch to the lowest and highest value within 1.5 times the interquartile region value from both the 25 and 75 percentile values respectively (Tukey's definition). The Y-axes have been split into 3 compartments according to the thresholds recommended by the World Health Organization Expert Working Group of GISRS for normal inhibition (NI) (A viruses <10-fold; B viruses <5-fold), reduced inhibition (RI) (A viruses 10- to 100-fold; B viruses 5- to 50-fold), and highly reduced inhibition (HRI) (A viruses >100-fold; B viruses >50-fold). For RI and HRI viruses that have been sequenced the determined AAS are shown; amino acid position numbering is A subtype- and B type-specific. All viruses were tested for susceptibility to oseltamivir and zanamivir but not all, including some variants, were tested against peramivir and laninamivir.

Of the 14,330 viruses tested, 113 (0.8%) exhibited RI or HRI by at least one NAI, a modest increase compared to the 2014–15 period (0.5%) (Fig. 2, Fig. 3A-D; Table 1, Table 2). NA sequence analysis revealed AASs in 102 of these 113 viruses. The presence of the identified NA AASs were confirmed in 76 matching clinical specimens and not detected in two; the remaining 24 clinical specimens were not available for analysis (Table 1, Table 2).

**Table 1:** Characteristics of 87 influenza type A viruses tested by WHO CCs that showed RI or HRI by at least one NAI with associated patient details.^a^

Virus	n	IC_50_ fold-change compared to reference median IC_50_ values b				NA substitution c		Patient setting	Antiviral treatment	Immuno-compromised
		Oseltamivir	Zanamivir	Peramivir	Laninamivir	Virus isolate	Clinical specimen			
A(H1N1)pdm09 n = 4544	64	**228 – 1317**	0.8–3.0	**119 – 417** (54)	1.0–4.4 (54)	H275Y	H275Y (53) Not available (11)	Community (22) Hospital (20) Unknown (22)	Oseltamivir (22) Peramivir (3) No (20) Unknown (19)	Yes (1) No (32) Unknown (31)
	8	**15**–**1070**	5.9–7.9	3.8–**255**	1.9–5.3	H275Y/H mix	H275Y/H mix (3) H275Y (3) Not available (2)	Community (7) Unknown (1)	Oseltamivir (4) Peramivir (1) No (1) Unknown (2)	No (7) Unknown (1)
	1	**2649**	5.2	**1427**	1.3	H275Y, G147R	H275Y, G147R	Hospital	Peramivir (1)	Yes
	1	**20,324**	8.3	**6270**	6.7	H275Y, I223K	H275Y, I223K	Hospital	Oseltamivir, peramivir and laninamivir (1)	Yes
	1	0.8	**10**	*n/t*^d^	*n/t*	I117R	I117R	Unknown	Unknown (1)	Unknown
	1	0.7	**20**	*n/t*	*n/t*	Q136R/Q mix	Not available	Hospital	No (1)	Unknown
	1	**22**	**21**	2.8	2.9	D151N/D mix	None	Community	No (1)	No
	2	**36–37**	**51–54**	**94–115**	**90–122**	S247R	S247R (1) Not available (1)	Unknown (2)	No (1) Unknown (1)	Unknown (2)
A(H3N2) n = 3714	1	**87**	**32**	**16**	9.0	Q391K	Q391K	Unknown	Unknown	Unknown
	7	**10–15**	3.9–5.8	0.9–2.6	1.3–2.5	None f	None	Unknown	Unknown	Unknown

a The number of viruses for which data was reported, if less than the number reported in column ‘n’, is shown in parentheses.

b RI and HRI fold-change values are displayed underlined and in bold typeface.

c Amino acid position numbering is A subtype specific.

d *n/t*: not tested.

f None: no amino acid substitutions (AASs) compared to the consensus sequence of viruses of the same type/subtype.

**Table 2:** Characteristics of 26 influenza type B viruses tested by WHO CCs that showed RI or HRI by at least one NAI with associated patient details a.

Virus	n	IC_50_ fold-change compared to reference median IC_50_ values b				NA substitution c		Patient setting	Antiviral treatment	Immuno-compromised
		Oseltamivir	Zanamivir	Peramivir	Laninamivir	Virus isolate	Clinical specimen			
B/Victoria- lineage n = 3190 d	1	**87**	**1220**	**17,724**	**701**	G104E	Not available	Unknown	Unknown	Unknown
	1	4.6	3.0	**59**	2.1	E105K/E	E105K/E	Unknown	Unknown	Unknown
	1	3.6	0.8	**76**	2.0	H134Y	H134Y	Community	Unknown	Unknown
	3	4.1–4.6	**121–159**	**100–131**	**49–53**	H134N	H134N	Hospital	No	Unknown
	1	1.9	2.2	**7.6**	2.6	I221T	None e	Hospital	No	No
	1	4.5	**6.1**	*n/t*^f^	*n/t*	G243S/G	Not available	Hospital	Unknown	No
	2	0.4–3.2	0.9–1.2	**23–54**	0.6–0.7	D432G	Not available	Unknown	Unknown	Unknown
	1	4.0	**14**	*n/t*	*n/t*	T106I, P165L	Not available	Hospital	Unknown	No
	1	3	**17**	*n/t*	*n/t*	K186R, I262T	Not available	Hospital	Unknown	No
	3	0.7–**13**	0.5–**5.8**	**5.1 – 8.9** (2)	0.7–1.1 (2)	None	None	Hospital (1) Unknown (3)	No (1) Unknown (3)	Yes(1) Unknown (3)
B/Yamagata- lineag n = 2882	1	0.8	0.5	**5.8**	0.9	None	Not available	Unknown	Unknown	Unknown
	1	**5.5**	1.5	1.2	2.1	K152N	K152N	Unknown	Unknown	Unknown
	5	4.8–**7.2**	4.0–**5.2**	**5.4 – 24** (4)	2.0–3.0 (4)	D197N	D197N (4) Not available (1)	Hospital (1) Unknown (4)	Unknown (5)	No (1) Unknown (4)
	1	2.0	2.0	**6.0**	3.0	I221V	I221V	Unknown	Unknown	Unknown
	3	4.6–**6.1**	1.0–1.3	**99–134**	0.9–1.0	H273Y	H273Y (2) Not available (1)	Community	No	No

a The number of viruses for which data was reported, if less than the number reported in column ‘n’, is shown within parentheses.

b RI and HRI fold-change values are displayed underlined and in bold typeface.

c Amino acid position numbering is B type specific.

d Includes 81 B/Yamagata-lineage haemagglutinin (HA) – B/Victoria-lineage neuraminidase (NA) reassortants; one of the reassortants had NA T106P + P165L AASs; no AASs were detected in two other reassortants displaying RI.

e None: no amino acid substitutions (AASs) compared to viruses with NI phenotype.

f *n/t*: not tested.

## 3 A(H1N1)pdm09 viruses showing RI or HRI

Of the 4544 A(H1N1)pdm09 viruses tested, 79 (1.7%) exhibited RI/HRI by at least one NAI (Table 1) which is slightly higher than in the previous season (0.5%; p < 0.001) (Fig. 2B).

The majority of these viruses exhibited RI/HRI by oseltamivir (n = 77; 15- to 20,324-fold) and peramivir (n = 65; 10- to 6270- fold), and most of them (n = 74) contained the NA H275Y AAS (Fig. 3A, Tables 1 and S1). These 74 viruses were collected in different parts of the world: Japan (n = 41), the United States of America (n = 19), China (n = 5), Norway (n = 3), Australia (n = 2), Singapore (n = 1) Malaysia (n = 1), Czechia (n = 1) and Oman (n = 1) (Table S1). Of the 74 viruses, 32 (43.2%) were from NAI-treated patients [oseltamivir (n = 26), peramivir (n = 5) or three NAIs (n = 1)]; 21 (28.4%) were from patients with no antiviral treatment, while 21 (28.4%) were from patients with unknown treatment histories (Tables 1 and S1). Eight of 74 viruses harboured mixed populations of H275Y variant and H275 wild type viruses (Table S1); five of these were from Japanese patients treated with oseltamivir (n = 4) or peramivir (n = 1). Notably, the two H275Y viruses displaying the highest fold increases in IC_50_ each contained an addition NA AAS. The first virus A/Hiroshima/13/2016 contained a novel NA dual AAS, H275Y + G147R, which conferred a 2649-fold increase in IC_50_. This virus was isolated from an immunocompromised patient who received prophylaxis with laninamivir and therapeutic treatment with peramivir (Takashita et al., 2016). The NA G147R AAS occurs at a low frequency in N1 subtype viruses (Hooper et al., 2015), and confers RI by oseltamivir and zanamivir in A(H5N1) viruses (Nguyen et al., 2013). More recently, it was shown to confer NA N1 receptor-binding activity, while retaining enzymatic activity and not affecting virus replication (Hooper and Bloom, 2013, Hooper et al., 2015). NA residue 147 is located in the 150-loop; it is possible that G147R alters the conformation of the 150-loop due to the larger size and positive charge of the side chain, thus adversely affecting the binding of NAIs and having a synergistic effect with H275Y. The second virus, A/Ibaraki/54/2016, was collected from a patient treated with oseltamivir, peramivir and laninamivir and contained NA H275Y + I223K dual AAS conferring 20,324-fold increase in IC_50_ (Tables 1 and S1). The combination of H275Y with I223K/R has been detected previously (Nguyen et al., 2012). It was shown that I223R narrows the NA active site pocket that accommodates the hydrophobic pentoxyl group of oseltamivir (Hurt et al., 2011, Van der Vries et al., 2012) and it is likely that I223K produces a similar effect.

In addition to RI by oseltamivir, two viruses (A/India/1819/2016 and A/Tennessee/24/2016) exhibited RI/HRI by the other three NAIs (Tables 1 and S1) and carried S247R (Table 1). This AAS was previously reported in two viruses, A/Sri Lanka/2356/2011 and A/Thailand/SirirajICRC_CBI_3/2009, and *in silico* studies predicted an adverse effect on oseltamivir and zanamivir binding (Mandal et al., 2017). Another AAS in the same residue, S247N, was previously shown to have a mild effect on inhibition by oseltamivir (5- to 8-fold) (Hurt et al., 2011). One virus, A/Bayern/151/2015 exhibited borderline RI (10-fold) by zanamivir and carried I117R (confirmed in the matching clinical specimen), which has not been described before.

Finally, two viruses exhibited RI and contained the NA AASs D151N/D and Q136R/Q previously associated with adaptation to cell culture (Tables 1 and S1).

## 4 A(H3N2) viruses displaying RI or HRI

As in previous seasons, the frequency of A(H3N2) viruses displaying RI/HRI remained very low (Fig. 2B). Of 3714 A(H3N2) viruses tested, 8 (0.2%) exhibited RI by one or more NAIs.

All eight viruses were collected in the United States of America and belonged to genetic group 3C.3a. A single virus, A/Indiana/29/2015, exhibited RI by oseltamivir, zanamivir, peramivir and borderline NI (9-fold) by laninamivir (Tables 1 and S1). The isolate and matching clinical specimen harboured NA Q391K AAS. The remaining seven viruses displayed borderline RI (10–15-fold) by oseltamivir. However, the RI phenotype of these viruses was not stable upon passage (data not shown). All viruses harboured NA K249E AAS, which has previously been seen in viruses displaying a borderline RI by oseltamivir (Sheu et al., 2008, Zhong et al., 2013).

## 5 B/Victoria-lineage viruses displaying RI or HRI

The number (n = 3190) of B/Victoria-lineage viruses tested during 2015–16 was four-times higher than during the previous season. However, the frequency of viruses displaying RI or HRI was slightly lower, 0.5% (15/3190) versus 0.7% (Fig. 2A–B).

One virus, B/Malaysia/0471/2016 exhibited HRI by all NAIs and harboured NA G104E AAS, which has not been described before, but neither patient information nor clinical specimen was available (Tables 2 and S2). A different AAS at the same position, G104R/G, was previously reported in a B/Victoria-lineage virus that displayed RI by peramivir (Hurt et al., 2016). A substitution at the neighbouring residue was detected in B/South Australia/48/2015 as a mixed population, NA E105K/E, associated with HRI by peramivir; the mixed population was also detected in the clinical specimen (Tables 2 and S2). This AAS has been linked to RI by NAIs in the past (Farrukee et al., 2015, Fujisaki et al., 2012). It has been suggested that NA AASs at residues 104 and 105 may weaken stability of the tetrameric NA and adversely affect binding of NAIs to the active site (Fujisaki et al., 2012), especially in B/Victoria-lineage viruses (Farrukee et al., 2015).

Three viruses displayed HRI by zanamivir and peramivir and borderline HRI by laninamivir (Fig. 3C) due to a novel NA H134N AAS, which was detected in these corresponding clinical specimens (Table S2) (Baranovich et al., 2017). These viruses were collected in two widely-dispersed provinces of Lao People's Democratic Republic (PDR) and no evidence of epidemiological links were found (Baranovich et al., 2017). One of the patients was hospitalized due to severe acute respiratory illness, and none of the patients had documented exposure to NAIs prior to specimen collection (Baranovich et al., 2017). NA H134 AAS may weaken binding of NAIs to the NA active site, especially for antivirals containing a guanidyl group, by affecting the conformation of the 150-loop. The H134N viruses replicated well in Normal Human Bronchial Epithelial (NHBE) cells and ferrets (Baranovich et al., 2017). The detection of influenza viruses displaying HRI by zanamivir in untreated patients is rare, which has been attributed to the minimalistic design of this NAI and infrequent use (McKimm-Breschkin, 2013). Another AAS at the same NA residue, H134Y, was detected in B/Christchurch/558/2015 (Tables 2 and S2) and associated with HRI by peramivir (76-fold) and NI by the other NAIs. This AAS has been previously reported in B/Victoria-lineage viruses showing RI by peramivir (Takashita et al., 2015b).

Two viruses exhibited RI/HRI by peramivir and contained the NA D432G AAS (Fig. 3C, Tables 2 and S2). This AAS was shown to confer RI/HRI by peramivir in B/Victoria-lineage viruses (Fujisaki et al., 2013, Leang et al., 2014). Unfortunately, the original specimens were not available for sequence analysis.

Additionally, three viruses that displayed RI by one or more NAIs showed no distinctive AASs in NA sequences (Tables 2 and S2). Two other viruses showed RI by zanamivir (14–17-fold) and contained the dual NA AAS of T106L + P165L and K186R + I262T, while a single virus showed borderline RI by zanamivir and contained the NA AAS G243S/G as a mixed population (Tables 2 and S2). Finally, a single virus (B/Darwin/83/2015) containing the NA AAS I221T exhibited RI by peramivir (∼8-fold); this AAS was not detected in the clinical specimen. NA I221T AAS has been shown to confer RI by peramivir in B/Victoria-lineage viruses (Leang et al., 2014).

## 6 B/Yamagata-lineage viruses showing RI or HRI

The number (n = 2882) of B/Yamagata-lineage viruses tested, as well as the frequency (0.4%; n = 11) of viruses displaying RI/HRI, was lower than in the previous season (Fig. 2A and B).

Three viruses isolated in different areas of Australia exhibited RI/HRI by peramivir and borderline RI by oseltamivir (Tables 2 and S2). These isolates and their respective clinical specimens contained NA H273Y AAS. This AAS has occasionally been seen in type B viruses from both lineages (Hurt et al., 2016, Meijer et al., 2014, Takashita et al., 2015b).

Five viruses containing D197N NA AAS were collected in widely-dispersed parts of the world (Tables 2 and S2). Consistent with previous reports (Hurt et al., 2016), this AAS conferred borderline RI by oseltamivir, zanamivir or peramivir (Table 2).

Three other viruses exhibited borderline RI by either oseltamivir or peramivir (Tables 2 and S2). The first virus contained the I221V NA AAS, previously reported in B/Victoria-lineage viruses circulating widely in North Carolina during 2011 (Garg et al., 2013, Sleeman et al., 2011). The second virus, B/Florida/05/2016 (isolate and clinical specimen) contained the K152N NA AAS (Tables 2 and S2), which has not been previously reported. Another AAS at the same residue, K152M, was reported in a B/Victoria-lineage virus exhibiting RI by oseltamivir (Hurt et al., 2016). The third virus had no apparent changes in the NA sequence (Table 2).

## 7 Frequency of RI and HRI conferring NA amino acid substitutions in sequence databases

The examination of NA sequences deposited in public sequence databases is a valuable adjunct to the drug susceptibility assessment. Sequences of seasonal influenza viruses collected between May 18, 2015 and May 22, 2016 were retrieved from two databases: the Global Initiative on Sharing All Influenza Data (GISAID) (www.gisaid.org) and National Centre for Biotechnology Information Influenza Virus Resource (NCBI-IVR) (www.ncbi.nlm.nih.gov/genomes/FLU/FLU.html). Sequences were curated by excluding those incomplete and/or duplicated (based on the strain designation), resulting in NA sequences for 13,484 influenza viruses (Fig. S1); approximately two-times the number during the 2014–15 reporting period (Hurt et al., 2016). The sequences were examined for substitutions previously reported to confer RI/HRI, as listed on the WHO GISRS website (http://www.who.int/influenza/gisrs_laboratory/antiviral_susceptibility/avwg2014_nai_substitution_table.pdf), as well as, additional AAS recently reported.

Based on the strain designation, we found that 8786 of the deposited sequences corresponded to viruses tested in NAI assay by WHO CCs, of which, 8673 belonged to viruses displaying NI (Fig. S1). Considering that some of them could harbour mixtures of wildtype and variant NA, and still exhibit a NI phenotype, it was prudent to inspect their sequences. Indeed, sequences of four A(H1N1)pdm09 virus isolates from Japan contained a NA H275Y/H mix; these viruses were collected from patients treated with oseltamivir (Table S3). In addition, A/Argentina/22/2015, contained NA I223T AAS, which conferred RI by oseltamivir in another A(H1N1)pdm09 virus (Takashita et al., 2015b). A group of 38 A(H1N1)pdm09 viruses contained NA AAS at E119 or Q136 (Table S3). The emergence of these variants could possibly be attributed to host-cell selection (Table S3). Although five of these sequences were reported as E119K, it is likely that an E119K/E mix was present in the virus isolate tested; clean E119K AAS, was shown to drastically diminish NA activity (Samson et al., 2014).

Three sequences of A(H3N2) viruses displaying NI contained the following AASs: E119V/E, R292K/R and Q136K/Q (Table S3). E119V is known to confer HRI by oseltamivir, while R292K confers HRI by oseltamivir/peramivir and RI by zanamivir/laninamivir. Q136K has been previously associated with virus adaptation to cell culture (Little et al., 2015).

Analysis of B/Victoria-lineage NA sequences revealed five viruses with the following AASs: G104E/G, E105K/E, G243D/G or G407S (Table S3). The latter AAS, was also found in three B/Yamagata-lineage NA sequences (Table S3). Moreover, two B/Yamagata-lineage NA sequences contained D197N or H134Y AAS. Notably, NA H134Y AAS in a B/Victoria-lineage virus (Table 2) conferred HRI by peramivir indicating a lineage-specific effect (Farrukee et al., 2015). One B/Victoria- and one B/Yamagata-lineage virus contained NA H273Q or H273N AAS, respectively. The fact that both viruses exhibited NI suggests that these AASs are not likely to affect drug susceptibility.

Overall, NA sequence analysis of the viruses tested in NAI assay showed good agreement between these two methods. Notably, sequencing allowed the detection of oseltamivir-resistant NA H275Y AAS subpopulations in virus isolates exhibiting NI and revealed selection of NA variants due to tissue-culture selection.

Lastly, analysis was carried out on 4698 (34.8%) NA sequences that belonged to viruses not tested by NAI assay. Of 2371 A(H1N1)pdm09 sequences, 40 (1.7%) contained H275Y, five of which were H275Y/H mixtures with one containing a dual AAS, H275Y/H + S247S/N (Table S4). A single virus, collected in the Russian Federation, carried NA S247R AAS that conferred RI/HRI by all four NAIs (Tables 1 and S4). The detection frequency of A(H1N1)pdm09 viruses carrying NA AASs associated with RI/HRI was similar among NAI assay-tested and sequence analysis-tested viruses (1.8% vs 1.7%, respectively).

Of 815 A(H3N2) NA sequences analysed, one (0.1%), collected in the United Kingdom, had E119V AAS (Table S4).

Among 1147 B/Victoria-lineage NA sequences, nine (0.7%) had the following AASs: E105K, D197N, I221V, K360E, D432G or N294S (Table S4). No substitutions were detected in NA sequences of 93 intra-lineage reassortants (B/Victoria-lineage NA). Of 365 B/Yamagata-lineage sequences, two (0.5%) contained D197N or N294S AASs (Table S4).

## 8 Concluding remarks

This is the fourth global update on seasonal influenza susceptibility to NAIs conducted by the WHO-AVWG of GISRS. The NAI assay data were generated by five WHO CCs on samples received from GISRS laboratories. In addition, NA sequences deposited in public databases by WHO CCs, NICs and other laboratories were interrogated to identify molecular changes known or suspected to alter susceptibility to NAIs.

Overall, the detection of seasonal influenza viruses exhibiting decreased susceptibility to NAIs remained low (∼0.09%). Consistent with the previous global updates, the frequency of viruses displaying RI or HRI was higher (∼1.8%) among A(H1N1)pdm09 viruses, with the majority of such viruses containing NA H275Y AAS. Available information indicates that many of these viruses were collected from patients not exposed to NAIs (Tables 1 and S1). This finding is in agreement with previous reports on the limited transmission of oseltamivir-resistant viruses containing NA H275Y AAS (Takashita et al., 2015b). Three other A(H1N1)pdm09 viruses, collected in widely-dispersed regions of the world, contained a rare NA S247R AAS that conferred RI/HRI against all four NAIs; the AAS was detected in the corresponding clinical specimens. Based on recent *in silico* studies (Mandal et al., 2017), this AAS was predicted to adversely affect binding of zanamivir and oseltamivir.

Another new NA H134N AAS was found in three B/Victoria-lineage viruses from Lao DPR that displayed HRI by zanamivir, peramivir and laninamivir (Baranovich et al., 2017). Presence of NA H134N AAS was confirmed in the respective clinical specimens, as for NA S247R AAS in A(H1N1)pdm09 viruses, thus ruling out their emergence due to tissue-culture selection.

Sequence analysis of those viruses displaying NI revealed that the NA changes detected fell into the following categories: 1) NA variants previously associated with RI/HRI that were present in a mix with wildtype (e.g. H275Y/H), but with too low amount of mutant to be detected by NAI assay; 2) NA variants previously shown to display borderline NI/RI (e.g. D197N); or 3) NA variants carrying changes associated with tissue-culture selection (e.g. E119K).

Likely due to a greater use of next-generation sequencing technologies by NICs globally, as well as, new initiatives (e.g. the Influenza Monitoring Vaccine Effectiveness (I-MOVE) program in Europe, Advanced Molecular Detection (AMD) in the USA) greater numbers of NA sequences are being deposited in databases. The number of sequences available for analysis during 2015–16 doubled compared to 2014–15; with a significant increase in the number of sequences from original clinical specimens. By comparing sequences generated from a virus isolate and the corresponding clinical specimen, we can determine if changes occur during virus isolation. This information improves the quality of drug susceptibility data by allowing removal of artefacts associated with tissue-culture selection.

While the list of NA changes reported to confer RI/HRI is growing (Hurt et al., 2016, Meijer et al., 2014, Takashita et al., 2015b), and the current list posted on the GISRS website needs updating, interpretation of sequencing data remains a challenge in the light of inadequate information on whether a particular NA change produces a consistent effect. Additional studies employing recombinant NA or reverse genetically engineered viruses may be needed to delineate the impact of the listed changes on phenotype assessed by NAI assay. Another limitation is the lack of information regarding the composition of virus populations; in virus preparations containing mixes, NI phenotype can be a reflection of the higher enzymatic activity of the wildtype subpopulation, which may obscure the presence of the dominant NA variant subpopulation (Mishin et al., 2014).

Based on available laboratory data, ∼99% of seasonal influenza viruses displayed NI by all NAIs, indicating that these drugs continue to be an appropriate choice for the treatment of influenza virus infection.

## Contributions

All WHO-AVWG Members and WHO headquarters and Regional Office Staff named were involved in the development of this to global update. LG and PJ drafted the manuscript and all authors contributed to editing the final manuscript. AH, S-KL, TGB, ET, TO generated and provided the NAI sensitivity data and molecular analysis. AM performed analysis of the data from the WHO CCs and drafted tables and figures.

## Disclaimer

The authors alone are responsible for the views expressed in this article and they do not necessarily represent the views, decisions or policies of the institutions with which they are affiliated.
