# Supplementary material for: Global update on the susceptibility of human influenza viruses to neuraminidase inhibitors, 2015–2016
Source: Antiviral Res. 2017 Oct;146:12–20. doi: 10.1016/j.antiviral.2017.08.004 (PMC5667636; doi:10.1016/j.antiviral.2017.08.004)
Supplement: Supplementary file 1 [file mmc1.pdf]

Fig S1. Data analysis flowchart

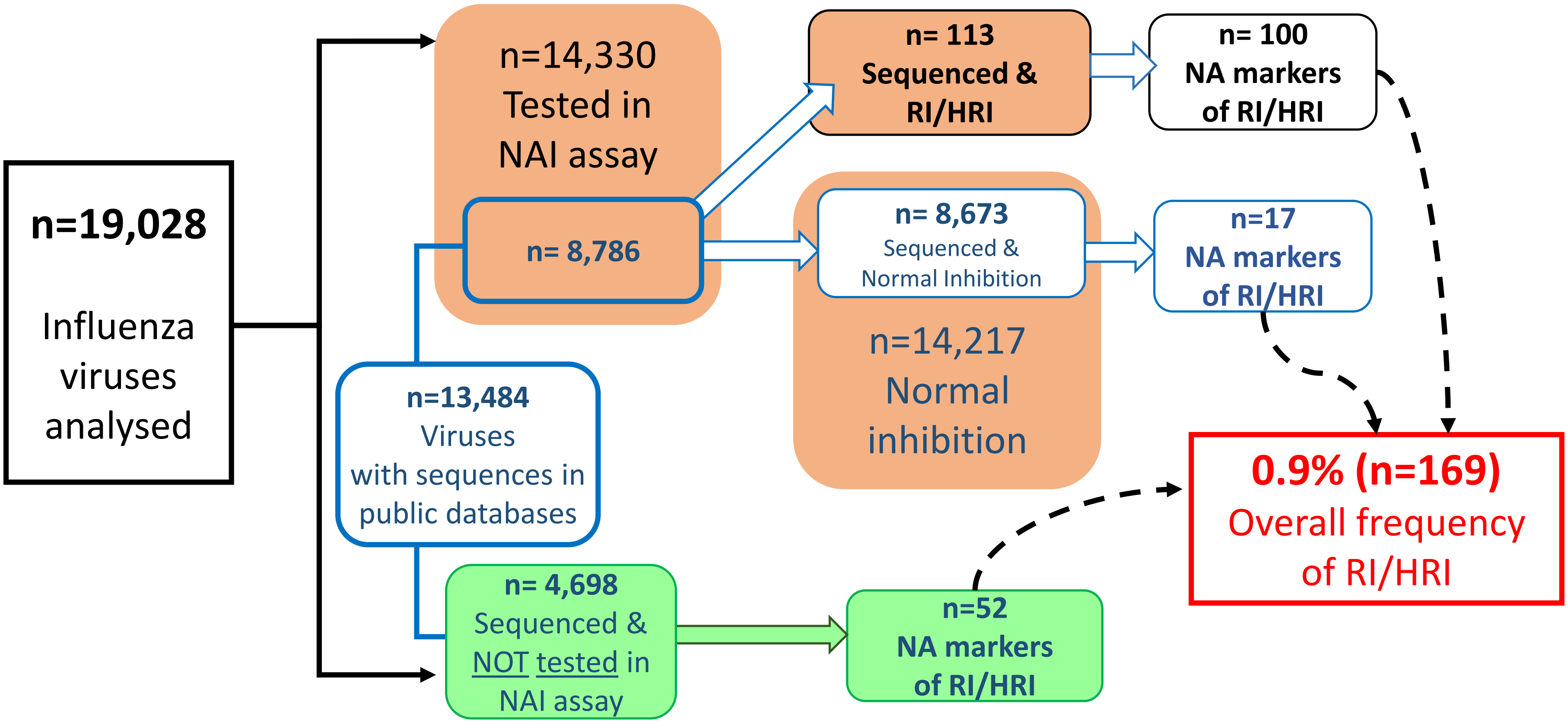

Figure S1: Data analysis flowchart. 19,028 influenza viruses were analysed during 2015-2016. Of those, 14,330 viruses were tested using neuraminidase (NA) inhibition (NAI) assay and 4,698 were tested only by NA sequence analysis. Of the 14,330 viruses tested in the NAI assay, 113 exhibited RI/HRI phenotype and n=100 carried NA AAS associated with RI/HRI. The vast majority of viruses tested in the NAI assay exhibited normal inhibition (n=14,217), and among the 8,673 that were sequenced, 17 carried NA markers associated with RI/HRI. Of the 4,698 viruses tested by NA sequence analysis, 52 carried NA AAS associated with RI/HRI. Overall, the frequency of viruses exhibiting or expected to exhibit RI/HRI was 0.9% (169/19,028) for the current 2015-2016 reporting period.
