# Supplementary material for: Global update on the susceptibility of human influenza viruses to neuraminidase inhibitors, 2015–2016
Source: Antiviral Res. 2017 Oct;146:12–20. doi: 10.1016/j.antiviral.2017.08.004 (PMC5667636; doi:10.1016/j.antiviral.2017.08.004)
Supplement: Supplementary file 2 [file mmc2.docx]

**Table S1:** Influenza A(H1N1)pdm09 and A(H3N2) virus isolates exhibiting RI or HRI by one or more NAIs (n=87).

|  | Sub-type | Strain Designation | WHO CC | Oseltamivir fold | Zanamivir fold | Peramivir fold | Laninamivir fold | NA  AAS ^a^ | original specimen | patient setting | antiviral  treatment | immuno- compromised | Date of collection  (d/m/y) |
| --- | --- | --- | --- | --- | --- | --- | --- | --- | --- | --- | --- | --- | --- |
| 1 | H1N1 | A/Ibaraki/54/2016 | Tokyo | **20,324** | 8.3 | **6,270** | 6.7 | H275Y, I223K | H275Y, I223K | Hospital | oseltamivir, peramivir, laninamivir | Yes | 07/03/16 |
| 2 | H1N1 | A/Hiroshima/13/2016 | Tokyo | **2,649** | 5.2 | **1,427** | 1.3 | H275Y, G147R | H275Y, G147R | Hospital | peramivir | Yes | 11/03/16 |
| 3 | H1N1 | A/Osaka/51/2016 | Tokyo | **1,317** | 1.2 | **294** | 2.2 | H275Y | H275Y | Community | peramivir | No | 05/03/16 |
| 4 | H1N1 | A/Shiga/24/2016 | Tokyo | **1,290** | 1.6 | **311** | 2.8 | H275Y | H275Y | Community | No | No | 01/04/16 |
| 5 | H1N1 | A/New York/38/2016 | Atlanta | **1,282** | 1.0 | **245** | 2.0 | H275Y | H275Y | UNK ^d^ | oseltamivir | UNK | 04/04/16 |
| 6 | H1N1 | A/Kumamoto/19/2016 | Tokyo | **1,268** | 1.1 | **278** | 3.1 | H275Y | H275Y | Community | oseltamivir | No | 25/02/16 |
| 7 | H1N1 | A/Tochigi/16151/2016 | Tokyo | **1,233** | 0.9 | **337** | 2.4 | H275Y | H275Y | Community | No | No | 04/03/16 |
| 8 | H1N1 | A/New York/37/2016 | Atlanta | **1,214** | 2.0 | **263** | 3.0 | H275Y | H275Y | UNK | oseltamivir | UNK | 24/03/16 |
| 9 | H1N1 | A/Kumamoto/25/2016 | Tokyo | **1,188** | 1.3 | **269** | 2.4 | H275Y | H275Y | Community | oseltamivir | No | 04/03/16 |
| 10 | H1N1 | A/Wakayama-C/89/2016 | Tokyo | **1,171** | 1.4 | **398** | 2.9 | H275Y | H275Y | Hospital | oseltamivir | UNK | 25/02/16 |
| 11 | H1N1 | A/Niigata-C/35/2016 | Tokyo | **1,164** | 1.2 | **319** | 4.2 | H275Y | H275Y | Community | UNK | No | 11/04/16 |
| 12 | H1N1 | A/Kanagawa/IC34/2016 | Tokyo | **1,159** | 1.0 | **306** | 3.7 | H275Y | H275Y | Community | No | No | 15/03/16 |
| 13 | H1N1 | A/Wakayama-C/97/2016 | Tokyo | **1,143** | 1.4 | **417** | 3.9 | H275Y | H275Y | Hospital | oseltamivir | UNK | 28/02/16 |
| 14 | H1N1 | A/Fujian-Sanyuan/SWL2262/2016 | Beijing | **1,132** | 3.0 | *n/t ^b^* | *n/t* | H275Y | N/A ^e^ | Hospital | UNK | No | 05/04/16 |
| 15 | H1N1 | A/Gunma/90/2016 | Tokyo | **1,127** | 1.4 | **236** | 1.4 | H275Y | H275Y | Community | No | No | 16/02/16 |
| 16 | H1N1 | A/Gunma/118/2016 | Tokyo | **1,112** | 0.9 | **265** | 1.7 | H275Y | H275Y | Community | No | No | 07/03/16 |
| 17 | H1N1 | A/New York/35/2016 | Atlanta | **1,080** | 1.0 | **181** | 2.0 | H275Y | H275Y | UNK | oseltamivir | UNK | 18/03/16 |
| 18 | H1N1 | A/Wyoming/16/2016 | Atlanta | **1,077** | 1.0 | **287** | 1.0 | H275Y | H275Y | UNK | UNK | UNK | 26/03/16 |
| 19 | H1N1 | A/Wakayama-C/99/2016 | Tokyo | **1,073** | 1.4 | **367** | 3.7 | H275Y | H275Y | Hospital | oseltamivir | UNK | 29/02/16 |
| 20 | H1N1 | A/Nara/25/2016 | Tokyo | **1,070** | 1.4 | **255** | 2.4 | H275Y/H mix | H275Y | Community | oseltamivir | No | 12/02/16 |
| 21 | H1N1 | A/Kagoshima/3193/2016 | Tokyo | **1,067** | 1.6 | **248** | 2.3 | H275Y | H275Y | Community | oseltamivir | No | 28/03/16 |
| 22 | H1N1 | A/Yamaguchi/24/2016 | Tokyo | **1,050** | 0.9 | **252** | 2.5 | H275Y | H275Y | Community | No | No | 13/02/16 |
| 23 | H1N1 | A/Michigan/65/2015 | Atlanta | **1,049** | 2.0 | **273** | 3.0 | H275Y | H275Y | UNK | oseltamivir | UNK | 24/10/15 |
| 24 | H1N1 | A/New York/33/2016 | Atlanta | **1,045** | 1.0 | **155** | 2.0 | H275Y | H275Y | UNK | UNK | UNK | 22/02/16 |
| 25 | H1N1 | A/Maryland/06/2016 | Atlanta | **1,042** | 1.0 | **243** | 2.0 | H275Y | H275Y | UNK | oseltamivir | UNK | 24/02/16 |
| 26 | H1N1 | A/Wakayama-C/101/2016 | Tokyo | **1,031** | 1.3 | **363** | 3.4 | H275Y | H275Y | Hospital | oseltamivir | UNK | 29/02/16 |
| 27 | H1N1 | A/Michigan/73/2016 | Atlanta | **1,024** | 2.0 | **249** | 3.0 | H275Y | H275Y | UNK | UNK | UNK | 18/04/16 |
| 28 | H1N1 | A/Chiba-C/1/2016 | Tokyo | **1,019** | 1.0 | **367** | 2.4 | H275Y | H275Y | Community | oseltamivir | No | 12/01/16 |
| 29 | H1N1 | A/Wakayama-C/90/2016 | Tokyo | **1,005** | 1.4 | **369** | 3.6 | H275Y | H275Y | Hospital | oseltamivir | UNK | 23/02/16 |
| 30 | H1N1 | A/Colorado/30/2015 | Atlanta | **971** | 1.0 | **238** | 2.0 | H275Y | H275Y | UNK | No | UNK | 14/12/15 |
| 31 | H1N1 | A/Georgia/31/2016 | Atlanta | **965** | 1.0 | **126** | 2.0 | H275Y | H275Y | UNK | UNK | UNK | 28/02/16 |
| 32 | H1N1 | A/Wakayama-C/102/2016 | Tokyo | **949** | 1.0 | **305** | 1.9 | H275Y | H275Y | Hospital | oseltamivir | UNK | 28/02/16 |
| 33 | H1N1 | A/Virginia/59/2016 | Atlanta | **948** | 2.0 | **269** | 3.0 | H275Y | H275Y | UNK | UNK | UNK | 15/05/16 |
| 34 | H1N1 | A/HAMAMATU-C/8/2016 | Tokyo | **948** | 1.4 | **238** | 1.9 | H275Y | H275Y | Hospital | peramivir | UNK | 06/01/16 |
| 35 | H1N1 | A/Saitama-C/10/2016 | Tokyo | **934** | 1.0 | **231** | 3.2 | H275Y | H275Y | Community | No | No | 07/02/16 |
| 36 | H1N1 | A/Yokohama/94/2016 | Tokyo | **934** | 1.1 | **261** | 1.8 | H275Y | H275Y | Community | No | No | 27/02/16 |
| 37 | H1N1 | A/New York/36/2016 | Atlanta | **924** | 1.0 | **229** | 3.0 | H275Y | H275Y | UNK | oseltamivir | UNK | 16/03/16 |
| 38 | H1N1 | A/TOCHIGI/16147/2016 | Tokyo | **913** | 1.0 | **234** | 2.9 | H275Y | H275Y | Community | No | No | 22/02/16 |
| 39 | H1N1 | A/North Carolina/42/2016 | Atlanta | **896** | 1.0 | **177** | 2.0 | H275Y | H275Y | UNK | No | UNK | 01/04/16 |
| 40 | H1N1 | A/Wakayama-C/100/2016 | Tokyo | **894** | 0.8 | **259** | 1.5 | H275Y | H275Y | Hospital | oseltamivir | UNK | 27/02/16 |
| 41 | H1N1 | A/Tokyo/15828/2016 | Tokyo | **879** | 1.1 | **208** | 2.9 | H275Y | N/A | Community | UNK | No | 28/02/16 |
| 42 | H1N1 | A/Fukushima/96/2016 | Tokyo | **877** | 1.7 | **247** | 2.9 | H275Y | H275Y | Community | No | No | 03/03/16 |
| 43 | H1N1 | A/Toyama/7/2016 | Tokyo | **876** | 1.8 | **201** | 3.0 | H275Y | H275Y | Community | No | No | 25/01/16 |
| 44 | H1N1 | A/New York/76/2016 | Atlanta | **850** | 2.0 | **253** | 2.0 | H275Y | H275Y | UNK | UNK | UNK | 26/04/16 |
| 45 | H1N1 | A/Toyama/4/2016 | Tokyo | **837** | 1.2 | **197** | 2.9 | H275Y | H275Y | Community | No | No | 16/01/16 |
| 46 | H1N1 | A/Tokyo/15806/2016 | Tokyo | **836** | 1.0 | **229** | 2.6 | H275Y | N/A | Community | UNK | No | 26/02/16 |
| 47 | H1N1 | A/Tottori/9/2016 | Tokyo | **834** | 1.0 | **287** | 3.9 | H275Y | H275Y | Hospital | oseltamivir | No | 14/02/16 |
| 48 | H1N1 | A/Pennsylvania/25/2016 | Atlanta | **818** | 2.0 | **238** | 2.0 | H275Y | H275Y | UNK | UNK | UNK | 21/02/16 |
| 49 | H1N1 | A/Shimane/40/2016 | Tokyo | **795** | 1.1 | **326** | 4.4 | H275Y | H275Y | Community | No | No | 11/03/16 |
| 50 | H1N1 | A/Yamaguchi/26/2016 | Tokyo | **793** | 0.8 | **277** | 1.4 | H275Y | H275Y | Hospital | peramivir | No | 18/02/16 |
| 51 | H1N1 | A/Brisbane/73/2016 | Melbourne | **793** | 2.0 | **150** | 3.0 | H275Y | H275Y | Hospital | No | No | 09/04/16 |
| 52 | H1N1 | A/Michigan/36/2016 | Atlanta | **781** | 2.0 | **224** | 2.0 | H275Y | H275Y | UNK | UNK | UNK | 17/02/16 |
| 53 | H1N1 | A/Illinois/44/2016 | Atlanta | **771** | 2.0 | **230** | 2.0 | H275Y | H275Y | UNK | oseltamivir | UNK | 07/03/16 |
| 54 | H1N1 | A/Washington/31/2016 | Atlanta | **767** | 1.0 | **239** | 2.0 | H275Y | N/A | UNK | No | UNK | 24/02/16 |
| 55 | H1N1 | A/Fujian-Tongan/ SWL153/2016 | Beijing | **716** | 2.0 | *n/t* | *n/t* | H275Y | N/A | Hospital | UNK | No | 19/01/16 |
| 56 | H1N1 | A/Aichi/96/2016 | Tokyo | **692** | 1.0 | **363** | 2.2 | H275Y | H275Y | Community | No | No | 12/04/16 |
| 57 | H1N1 | A/Hunan-Yueyanglou/SWL119/2016 | Beijing | **618** | 1.4 | *n/t* | *n/t* | H275Y | N/A | Hospital | UNK | No | 07/01/16 |
| 58 | H1N1 | A/Singapore/TT1275/2015 | Melbourne | **594** | 1.0 | **119** | 2.0 | H275Y | H275Y | Hospital | No | Yes | 09/10/15 |
| 59 | H1N1 | A/Malaysia/3039/2015 | Melbourne | **557** | 1.0 | **123** | 2.0 | H275Y | N/A | Hospital | No | No | 20/10/15 |
| 60 | H1N1 | A/Fujian-Sanyuan/SWL236/2016 | Beijing | **520** | 0.8 | *n/t* | *n/t* | H275Y | N/A | Hospital | UNK | No | 14/01/16 |
| 61 | H1N1 | A/Sydney/185/2015 | Melbourne | **477** | 1.0 | **141** | 1.0 | H275Y | H275Y | Hospital | oseltamivir | No | 10/08/15 |
| 62 | H1N1 | A/Oman/1726/2016 | London | **459** | 1.4 | *n/t* | *n/t* | H275Y | N/A | UNK | UNK | UNK | 09/02/16 |
| 63 | H1N1 | A/Czech Republic/11/2016 | London | **392** | 1.9 | *n/t* | *n/t* | H275Y | N/A | Hospital | oseltamivir | UNK | 28/01/16 |
| 64 | H1N1 | A/Norway/2298/2016 | London | **389** | 1.3 | *n/t* | *n/t* | H275Y | H275Y | UNK | UNK | UNK | 21/03/16 |
| 65 | H1N1 | A/Norway/2036/2016 | London | **353** | 1.2 | *n/t* | *n/t* | H275Y | H275Y | UNK | oseltamivir | UNK | 10/03/16 |
| 66 | H1N1 | A/Niigata/50/2016 | Tokyo | **307** | 1.1 | **61** | 2.3 | H275Y/H mix | H275Y | Community | oseltamivir | No | 19/01/16 |
| 67 | H1N1 | A/Norway/2914/2015 | London | **268** | 1.0 | *n/t* | *n/t* | H275Y | H275Y | UNK | UNK | UNK | 14/12/15 |
| 68 | H1N1 | A/Gunma/4/2016 | Tokyo | **249** | 1.2 | **53** | 2.3 | H275Y/H mix | H275Y/H mix | Community | oseltamivir | No | 12/01/16 |
| 69 | H1N1 | A/Guangdong-Duanzhou/SWL1325/2016 | Beijing | **228** | 0.8 | n/t | n/t | H275Y | N/A | Hospital | UNK | No | 19/04/16 |
| 70 | H1N1 | A/Tokyo/EH7/2016 | Tokyo | **221** | 0.8 | **76** | 2.9 | H275Y/H mix | H275Y/H mix | Community | oseltamivir | No | 05/02/16 |
| 71 | H1N1 | A/Kumamoto/21/2016 | Tokyo | **197** | 1.9 | **43** | 3.9 | H275Y/H mix | H275Y | Community | No | No | 23/02/16 |
| 72 | H1N1 | A/India/1819/2016 | Atlanta | **37** | **54** | **115** | **122** | S247R | N/A | UNK | UNK | UNK | 18/02/16 |
| 73 | H1N1 | A/Tennessee/24/2016 | Atlanta | **36** | **51** | **94** | **90** | S247R | S247R | UNK | No | UNK | 19/04/16 |
| 74 | H1N1 | A/Yamagata/7/2016 | Tokyo | **34** | 7.9 | **19** | 5.3 | H275Y/H mix | H275Y/H mix | Community | peramivir | No | 19/01/16 |
| 75 | H1N1 | A/Quebec/RV1424/2016 | Atlanta | **34** | 1.0 | **10** | 2.0 | H275Y/H mix | N/A | UNK | UNK | UNK | 22/02/16 |
| 76 | H1N1 | A/Aomori/5/2016 | Tokyo | **22** | **21** | 2.8 | 2.9 | D151N/D mix | None | Community | No | No | 16/01/16 |
| 77 | H1N1 | A/Tokyo/15768/2016 | Tokyo | **15** | 5.9 | 3.8 | 1.9 | H275Y/H mix | N/A | Community | UNK | No | 09/02/16 |
| 78 | H1N1 | A/Bayern/151/2015 | London | 0.8 | **10** | *n/t* | *n/t* | I117R | I117R | UNK | UNK | UNK | 26/12/15 |
| 79 | H1N1 | A/Odessa/699/2016 | London | 0.7 | **20** | *n/t* | *n/t* | Q136R/Q mix | N/A | Hospital | No | UNK | 08/02/16 |
|  |  |  |  |  |  |  |  |  |  |  |  |  |  |
| 1 | H3N2 | A/Indiana/29/2015 | Atlanta | **87** | **32** | **16** | 9.0 | Q391K | N/A | UNK | UNK | UNK | 27/11/15 |
| 2 | H3N2 | A/Michigan/47/2016 | Atlanta | **15** | 3.9 | 2.6 | 1.9 | None ^c^ | None | UNK | UNK | UNK | 07/03/16 |
| 3 | H3N2 | A/North Dakota/26/2016 | Atlanta | **14** | 3.9 | 1.4 | 1.3 | None | None | UNK | UNK | UNK | 22/03/16 |
| 4 | H3N2 | A/Florida/13/2016 | Atlanta | **13** | 5.0 | 1.4 | 1.9 | None | None | UNK | UNK | UNK | 01/02/16 |
| 5 | H3N2 | A/New York/21/2016 | Atlanta | **12** | 5.8 | 1.9 | 2.5 | None | None | UNK | UNK | UNK | 13/02/16 |
| 6 | H3N2 | A/Florida/52/2016 | Atlanta | **11** | 3.6 | 0.9 | 1.4 | None | None | UNK | UNK | UNK | 19/02/16 |
| 7 | H3N2 | A/Florida/22/2016 | Atlanta | **11** | 5.3 | 1.5 | 2.4 | None | None | UNK | UNK | UNK | 24/01/16 |
| 8 | H3N2 | A/New York/18/2016 | Atlanta | **10** | 4.8 | 1.1 | 1.9 | None | None | UNK | UNK | UNK | 05/02/16 |

^a^ Amino acid position numbering is A subtype specific. AAS, amino acid substitution.

^b^ n/t: not tested.

^c^ None: no amino acid substitutions compared to viruses with normal inhibition phenotype.

^d^ UNK: unknown patient setting or treatment history.

^e^ N/A: original specimen not available.
