# Supplementary material for: Global update on the susceptibility of human influenza viruses to neuraminidase inhibitors, 2015–2016
Source: Antiviral Res. 2017 Oct;146:12–20. doi: 10.1016/j.antiviral.2017.08.004 (PMC5667636; doi:10.1016/j.antiviral.2017.08.004)
Supplement: Supplementary file 3 [file mmc3.docx]

**Table S2: Total numbers of influenza A (H1N1)pdm09 viruses carrying NA AAS H275Y organised by country of origin**

1. **Viruses exhibiting RI/HRI in the NAI assay and carrying NA AAS H275Y**

| WHO region | Country | No of viruses tested in the NAI assay | No of viruses displaying RI/HRI phenotype because NA AAS H275Y (%) | Patient antiviral exposure (n) | | |
| --- | --- | --- | --- | --- | --- | --- |
|  |  |  |  | **yes** | **no** | **unknown** |
| Western Pacific | Australia | 434 | 2 (0.5%) | 1 | 1 | 0 |
| Americas | Canada | 26 | 1 (3.8%) | 0 | 0 | 1 |
| Western Pacific | China | 678 | 5 (0.7%) | 0 | 0 | 5 |
| Europe | Czechia | 16 | 1 (6.3%) | 1 | 0 | 0 |
| Western Pacific | **Japan** | 342 | **41 (12%)** | 22 | 15 | 4 |
| Western Pacific | Malaysia | 43 | 1 (2.3%) | 0 | 1 | 0 |
| Europe | **Norway** | 20 | **3 (15%)** | 1 | 0 | 2 |
| Eastern Mediterranean | Oman | 26 | 1 (3.8%) | 0 | 0 | 1 |
| Western Pacific | Singapore | 40 | 1 (2.5%) | 0 | 1 | 0 |
| Americas | USA | 1,262 | 18 (1.4%) | 7 | 3 | 8 |
| Various regions | Other countries | 1,683 | 0 (0.0%) | 0 | 0 | 0 |
|  | **Total** | **4,544** | **74 (1.6%)** | **32** | **21** | **21** |

1. **All A(H1N1)pdm09 viruses carrying NA AAS H275Y**

| Country | No. of A(H1N1)pdm09 viruses tested using the method: | | | | No. of viruses carrying NA AAS H275Y detected using the method: | | |
| --- | --- | --- | --- | --- | --- | --- | --- |
|  | **Total (NAI assay and/or NA sequencing analysis)** | **NAI assay and NA sequencing analysis assay ^a^** | **NAI assay only** | **NA sequencing**  **only ^b^** | **NAI assay**  **(%)^c^** | **NA sequencing**  **(%)^c^** | **Total No by both methods (%)^c^** |
| Australia | 435 | 118 | 316 | 1 | 2 (0.5%) | 0 (0%) | 2 (0.5%) |
| Belgium | 3 | 2 | 0 | 1 | 0 (0%) | 1 (100%) | 1 (33.3%) |
| Brazil | 124 | 22 | 1 | 101 | 0 (0%) | 1 (1%) | 1 (0.8%) |
| Canada | 29 | 26 | 0 | 3 | 1 (3.8%) | 0 (0%) | 1 (3.4%) |
| China | 735 | 111 | 567 | 57 | 5 (0.7%) | 0 (0%) | 5 (0.7%) |
| Czechia | 19 | 14 | 2 | 3 | 1 (6.3%) | 1 (33.3%) | 2 (10.5%) |
| Egypt | 83 | 22 | 0 | 61 | 0 (0%) | 2 (3.3%) | 2 (2.4%) |
| France | 181 | 10 | 0 | 171 | 0 (0%) | 1 (0.6%) | 1 (0.6%) |
| Greece | 55 | 22 | 0 | 33 | 0 (0%) | 1 (3%) | 1 (1.8%) |
| Japan | 384 | 224 | 118 | 42 | **41 (12%)** | **8 (19%)^d^** | **49 (12.8%)** |
| Malaysia | 43 | 8 | 35 | 0 | 1 (2.3%) | 0 (0%) | 1 (2.3%) |
| Norway | 50 | 19 | 1 | 30 | **3 (15%)** | **8 (26.7%)** | **11 (22%)** |
| Oman | 31 | 22 | 4 | 5 | 1 (3.8%) | 0 (0%) | 1 (3.2%) |
| Russian Federation | 455 | 57 | 6 | 392 | 0 (0%) | 8 (2%) | 8 (1.8%) |
| Singapore | 158 | 37 | 3 | 118 | 1 (2.5%) | 1 (0.8%) | 2 (1.3%) |
| Spain | 66 | 36 | 3 | 27 | 0 (0%) | 2 (7.4%) | 2 (3%) |
| United Kingdom | 357 | 1 | 0 | 356 | 0 (0%) | 4 (1.1%) | 4 (1.1%) |
| USA | 1,394 | 1,237 | 25 | 132 | 18 (1.4%) | 2 (1.5%) | 20 (1.4%) |
| Other countries | 2,313 | 1,256 | 219 | 838 | 0 (0%) | 0 (0%) | 0 (0%) |
| Total | **6,915** | **3,244** | **1,300** | **2,371** | **74 (1.6%)** | **40 (1.7%)** | **114 (1.6%)** |

^a^ All A(H1N1)pdm09 viruses tested in the NAI assay that showed RI/HRI were sequenced.

^b^ These numbers represent viruses that were not characterized in the NAI assay and only NA sequence are available

^c^ Numbers in parenthesis show the detection frequency of H275Y calculated by each method

^d^ Four of these viruses corresponded to isolates containing H275Y/H mixes and displaying normal inhibition in the NAI assay.
