# Supplementary material for: Global update on the susceptibility of human influenza viruses to neuraminidase inhibitors, 2015–2016
Source: Antiviral Res. 2017 Oct;146:12–20. doi: 10.1016/j.antiviral.2017.08.004 (PMC5667636; doi:10.1016/j.antiviral.2017.08.004)
Supplement: Supplementary file 4 [file mmc4.docx]

**Table S3:** Influenza B/Victoria/2/87- lineage and Influenza B/Yamagata/16/88- lineage viruses exhibiting RI or HRI by one or more NAIs

|  | Lineage | Strain Designation | WHO CC | Oseltamivir fold | Zanamivir fold | Peramivir fold | Laninamivir fold | NA  AAS ^a^ | Original specimen | patient setting | antiviral treatment | immuno- compromised | Date of collection (d/m/y) |
| --- | --- | --- | --- | --- | --- | --- | --- | --- | --- | --- | --- | --- | --- |
| 1 | B Vic | B/Malaysia/0471/2016 | Melbourne | **87** | **1,220** | **17,724** | **701** | G104E | N/A^e^ | UNK ^f^ | UNK | UNK | 21/01/16 |
| 2 | B Vic | B/Laos/0406/2016 | Atlanta | 4.1 | **155** | **128** | **53** | H134N | H134N | UNK | UNK | UNK | 09/02/16 |
| 3 | B Vic | B/Laos/0654/2016 | Atlanta | 4.1 | **121** | **100** | **49** | H134N | H134N | UNK | UNK | UNK | 25/02/16 |
| 4 | B Vic | B/Laos/0525/2016 | Atlanta | 4.6 | **159** | **131** | **50** | H134N | H134N | UNK | UNK | UNK | 15/02/16 |
| 5 | B Vic | B/Christchurch/558/2015 | Melbourne | 3.6 | 0.8 | **76** | 2.0 | H134Y | H134Y | Community | UNK | UNK | 14/08/15 |
| 6 | B Vic | B/South Australia/48/2015 | Melbourne | 4.6 | 3.0 | **59** | 2.1 | E105K/E | E105K/E | UNK | UNK | UNK | 30/05/15 |
| 7 | B Vic | B/Darwin/83/2015 | Melbourne | 1.9 | 2.2 | **7.6** | 2.6 | I221T | None | Hospital | No | No | 17/10/15 |
| 8 | B Vic | B/Neimenggu-Yuquan/1171/2016 | Beijing | 4.5 | **6.1** | *n/t ^b^* | *n/t* | G243G/S | N/A | Hospital | UNK | No | 24/02/16 |
| 9 | B Vic | B/Idaho/19/2015 | Atlanta | 0.4 | 1.2 | **54** | 0.7 | D432G | N/A | UNK | UNK | UNK | 05/06/15 |
| 10 | B Vic | B/Brisbane/240/2015 | Melbourne | 3.2 | 0.9 | **23** | 0.6 | D432G | N/A | Hospital | No | Yes | 03/05/16 |
| 11 | B Vic | B/Neimenggu-Yuquan/11363/2016 | Beijing | 3.1 | **17** | *n/t* | *n/t* | K186R,I262T | N/A | Hospital | UNK | No | 14/03/16 |
| 12 | B Vic NA (HA/Yam) ^c^ | B/Anhui-Dangtu/1427/2016 | Beijing | 4.0 | **14** | *n/t* | *n/t* | T106I,P165L | N/A | Hospital | UNK | No | 29/12/15 |
| 13 | B Vic | B/Oman/7822/2015 | London | 3.4 | **5.8** | *n/t* | *n/t* | None ^d^ | N/A | UNK | UNK | UNK | 08/08/15 |
| 14 | B Vic NA (HA/Yam) | B/Pakistan/425/2015 | Atlanta | **13** | 0.5 | **5.1** | 0.7 | None | None | UNK | UNK | UNK | 08/12/15 |
| 15 | B Vic NA (HA/Yam) | B/New York/23/2015 | Atlanta | 0.7 | 0.8 | **8.9** | 1.1 | None | None | UNK | UNK | UNK | 26/12/15 |
|  |  |  |  |  |  |  |  |  |  |  |  |  |  |
| 1 | B Yam | B/Sydney/53/2015 | Melbourne | **6.1** | 1.6 | **119** | 1.0 | H273Y | N/A | Community | No | No | 08/06/15 |
| 2 | B Yam | B/South Australia/ 136/2015 | Melbourne | **5.8** | 1.3 | **134** | 0.9 | H273Y | H273Y | Community | No | No | 07/10/15 |
| 3 | B Yam | B/Perth/136/2015 | Melbourne | 4.6 | 1.0 | **99** | 1.0 | H273Y | H273Y | Community | No | No | 29/07/15 |
| 4 | B Yam | B/Gorbea/75877/2015 | Atlanta | **6.9** | 4.0 | **24** | 3.3 | D197N | D197N | UNK | UNK | UNK | 21/10/15 |
| 5 | B Yam | B/Santiago/75552/2015 | Atlanta | **7.2** | 4.2 | **18** | 2.0 | D197N | D197N | UNK | UNK | UNK | 22/10/15 |
| 6 | B Yam | B/Bangladesh/6263/2015 | Atlanta | **5.5** | 4.4 | **5.8** | 3.0 | D197N | D197N | UNK | UNK | UNK | 14/11/15 |
| 7 | B Yam | B/Utah/15/2016 | Atlanta | 4.8 | **5.2** | **5.4** | 3.0 | D197N | D197N | UNK | UNK | UNK | 07/01/16 |
| 8 | B Yam | B/Hubei-Wuchang/ 181/2016 | Beijing | **5.9** | 3.9 | *n/t* | *n/t* | D197N | N/A | Hospital | UNK | No | 01/02/16 |
| 9 | B Yam | B/Florida/05/2016 | Atlanta | **5.5** | 1.5 | 1.2 | 2.1 | K152N | K152N | UNK | UNK | UNK | 10/01/16 |
| 10 | B Yam | B/Malaysia/2104/2015 | Melbourne | 0.8 | 0.5 | **5.8** | 0.9 | None | N/A | UNK | UNK | UNK | 12/06/15 |
| 11 | B Yam | B/Wisconsin/10/2016 | Atlanta | 2.0 | 2.0 | **6.0** | 3.0 | I221V | I221V | UNK | UNK | UNK | 19/02/16 |

^a^ Amino acid position numbering is B subtype specific (same NA numbering for B/Victoria- and B/Yamagata-lineage). AAS, amino acid substitution.

^b^ *n/t:* not tested.

^c^ B Vic NA (HA/Yam): these viruses are B/Yamagata-lineage haemagglutinin (HA) – B/Victoria-lineage neuraminidase (NA) reassortants.

^d^ None: no amino acid substitutions compared to consensus sequence of viruses with normal inhibition phenotype.

^e^ N/A: original specimen not available.

^f^: UNK: unknown patient setting or treatment history.
