# Supplementary material for: Global update on the susceptibility of human influenza viruses to neuraminidase inhibitors, 2015–2016
Source: Antiviral Res. 2017 Oct;146:12–20. doi: 10.1016/j.antiviral.2017.08.004 (PMC5667636; doi:10.1016/j.antiviral.2017.08.004)
Supplement: Supplementary file 5 [file mmc5.docx]

**Table S4:** Influenza A and B viruses harboring NA changes and displaying normal inhibition in the NAI assay

|  | Strain designation | WHO CC | A subtype  /B lineage | Passage details/history ^a^ | NA  AAS ^b^ | NA GISAID Acc. No. | Original specimen |
| --- | --- | --- | --- | --- | --- | --- | --- |
| 1 | A/Ukraine/7029/2016 | London | A(H1N1)pdm09 | P2/C1 | E119G/E | EPI773937 | UNK |
| 2 | A/Talca/20692/2016 | Atlanta | A(H1N1)pdm09 | E2/E1 | E119G/E G147R | EPI832564 | E119 |
| 3 | A/California/102/2015 | Atlanta | A(H1N1)pdm09 | E3 | E119K/E ^c^ | EPI695521 | E119 |
| 4 | A/Colorado/17/2015 | Atlanta | A(H1N1)pdm09 | C1 | E119K/E | EPI685450 | E119 |
| 5 | A/Costa Rica/7886/2015 | Atlanta | A(H1N1)pdm09 | C2 | E119K/E | EPI756355 | E119 |
| 6 | A/Michigan/07/2016 | Atlanta | A(H1N1)pdm09 | C1 | E119K/E | EPI732903 | E119 |
| 7 | A/Denmark/49/2015 | London | A(H1N1)pdm09 | SIAT2/C2/C1 | E119K/E | EPI697694 | UNK |
| 8 | A/Fukuoka/15034/2015 | Tokyo | A(H1N1)pdm09 | C3 +1 | E119K/E | EPI706472 | UNK |
| 9 | A/Fukushima/74/2015 | Tokyo | A(H1N1)pdm09 | C2 +1 | E119K/E | EPI673545 | UNK |
| 10 | A/Fukushima/76/2015 | Tokyo | A(H1N1)pdm09 | C1 | E119K/E | EPI673565 | UNK |
| 11 | A/Guangdong-chancheng/swl1361/2016 | Beijing | A(H1N1)pdm09 | C2+C1 | E119K/E | EPI777754 | UNK |
| 21 | A/Jordan/10118/2016 | London | A(H1N1)pdm09 | Cx/C1 | E119K/E | EPI718205 | UNK |
| 13 | A/Kanagawa/ic10/2016 | Tokyo | A(H1N1)pdm09 | C0 +1 | E119K/E | EPI721750 | UNK |
| 14 | A/Kanagawa/ic24/2016 | Tokyo | A(H1N1)pdm09 | C0 +1 | E119K/E | EPI731520 | UNK |
| 15 | A/Nagano/2118/2016 | Tokyo | A(H1N1)pdm09 | C1 +1 | E119K/E | EPI731484 | UNK |
| 16 | A/Nagano/2536/2015 | Tokyo | A(H1N1)pdm09 | C1 +1 | E119K/E | EPI678226 | UNK |
| 17 | A/Ontario/rv0023/2016 | Atlanta | A(H1N1)pdm09 | X1 | E119K/E | EPI742124 | UNK |
| 18 | A/Ontario/rv1528/2016 | Atlanta | A(H1N1)pdm09 | X1 | E119K/E | EPI791922 | UNK |
| 19 | A/Osaka-c/39/2016 | Tokyo | A(H1N1)pdm09 | C1 +1 | E119K/E | EPI831444 | UNK |
| 20 | A/Shanghai-Fengxian/swl11127/2015 | Beijing | A(H1N1)pdm09 | E2 | E119K/E | EPI713377 | UNK |
| 21 | A/Shanghai-Putuo/swl1996/2015 | Beijing | A(H1N1)pdm09 | E3 | E119K/E | EPI713341 | UNK |
| 22 | A/Taiwan/533/2016 | Tokyo | A(H1N1)pdm09 | C3 +1 | E119K/E | EPI813635 | UNK |
| 23 | A/Tokyo/eh12/2016 | Tokyo | A(H1N1)pdm09 | C0 +1 | E119K/E | EPI731506 | UNK |
| 24 | A/Brisbane/137/2015 | Melbourne | A(H1N1)pdm09 | E4 | E119K | EPI746389 | UNK |
| 25 | A/Shanghai-Huangpu/ SWL12017/2015 | Beijing | A(H1N1)pdm09 | E2 | E119K | EPI697215 | UNK |
| 26 | A/Fukuoka/15036/2015 | Tokyo | A(H1N1)pdm09 | C3 | E119K | EPI699909 | UNK |
| 27 | A/Manitoba/RV1967/2016 | Atlanta | A(H1N1)pdm09 | X1 | E119K | EPI791970 | UNK |
| 28 | A/Denmark/43/2015 | London | A(H1N1)pdm09 | C3/C1 | E119K | EPI717660 | UNK |
| 29 | A/Ishikawa/54/2016 | Tokyo | A(H1N1)pdm09 | C1 | H275Y/H | EPI745952 | H275Y/H |
| 30 | A/Saitama-C/9/2016 | Tokyo | A(H1N1)pdm09 | C2 | H275Y/H | EPI721756 | H275Y/H |
| 31 | A/Yokohama/87/2016 | Tokyo | A(H1N1)pdm09 | C1 | H275Y/H | EPI722932 | H275Y/H |
| 32 | A/Ibaraki/27/2016 | Tokyo | A(H1N1)pdm09 | C1 +1 | H275Y/H | EPI764241 | H275Y/H |
| 33 | A/Argentina/22/2015 | Atlanta | A(H1N1)pdm09 | C1 | I223T ^c^ | EPI651822 | UNK |
| 34 | A/Kobe/248/2016 | Tokyo | A(H1N1)pdm09 | C1 +1 | Q136K/Q ^d^ | EPI823477 | UNK |
| 35 | A/Israel/Q-363/2015 | London | A(H1N1)pdm09 | C1/C1 | Q136K/Q | EPI717676 | UNK |
| 36 | A/Yokohama/92/2015 | Tokyo | A(H1N1)pdm09 | C1 +1 | Q136R/Q | EPI673553 | UNK |
| 37 | A/Miyagi/27/2016 | Tokyo | A(H1N1)pdm09 | C1 +1 | Q136R/Q | EPI769345 | UNK |
| 38 | A/Hokkaido/7/2016 | Tokyo | A(H1N1)pdm09 | C1 +1 | Q136R/Q | EPI770083 | UNK |
| 39 | A/Iwate/27/2016 | Tokyo | A(H1N1)pdm09 | C1 +1 | Q136R/Q | EPI769349 | UNK |
|  |  |  |  |  |  |  |  |
| 1 | A/Victoria/2502/2015 | Melbourne | H3N2 | SIAT2 | E119V/E | EPI652507 | UNK |
| 2 | A/Odessa/700/2016 | London | H3N2 | SIAT2/SIAT1 | R292K/R | EPI773642 | UNK |
| 3 | A/Hyogo/2001/2015 | Tokyo | H3N2 | C2 +SIAT1 | Q136K/Q D151D/N | EPI678208 | UNK |
|  |  |  |  |  |  |  |  |
| 1 | B/Hamamatu-C/78/2016 | Tokyo | B Victoria | C2 | G104E/G | EPI756001 | UNK |
| 2 | B/Iwate/1/2016 | Tokyo | B Victoria | C1 +1 | E105K/E | EPI636464 | UNK |
| 3 | B/Bayern/14/2016 | London | B Victoria | C1/C1 | G243D/G ^e^ | EPI717297 | UNK |
| 4 | B/Newcastle/1005/2015 | Melbourne | B Victoria | C1 | G407S | EPI758810 | UNK |
|  |  |  |  |  |  |  |  |
| 1 | B/Mozambique/IR981/2015 | London | B Yamagata | cs/C2 | H134Y ^f^ | EPI630512 | UNK |
| 2 | B/Singapore/Gp702/2015 | Melbourne | B Yamagata | C1 | D197N | EPI820247 | UNK |
| 3 | B/Alaska/02/2016 | Atlanta | B Yamagata | C1 | G407S | EPI717993 | G407S |
| 4 | B/Wisconsin/21/2016 | Atlanta | B Yamagata | C1 | G407S | EPI776512 | G407S |
| 5 | B/Wisconsin/42/2016 | Atlanta | B Yamagata | C1 | G407S | EPI664177 | G407S |

*Note:* passage of virus tested and virus sequence may differ in some instances.

^a^ Passage as shown in the sequence databases.

^b^ NA numbering is subtype-specific. NA amino acid substitutions (AAS) associated with reduced inhibition, as listed in the summary table on the WHO GISRS website <http://www.who.int/influenza/gisrs_laboratory/antiviral_susceptibility/avwg2014_nai_substitution_table.pdf>

^c^ HRI to laninamivir and oseltamivir [Samson et al, 2014] and RI to oseltamivir [Takashita et al, 2015b]

^d^ HRI against zanamivir and peramivir and RI against laninamivir [Meijer et al, 2014].

^e^ In this study

^f^ H134Y/H showed RI against peramivir [Takashita et al, 2015b]
