# Supplementary material for: Global update on the susceptibility of human influenza viruses to neuraminidase inhibitors, 2015–2016
Source: Antiviral Res. 2017 Oct;146:12–20. doi: 10.1016/j.antiviral.2017.08.004 (PMC5667636; doi:10.1016/j.antiviral.2017.08.004)
Supplement: Supplementary file 6 [file mmc6.docx]

**Table S5:** Influenza A and B viruses (n=52) with unavailable NI data carrying NA substitutions associated to HRI/RI

|  | Designation | Submitting lab | A subtype  /B lineage | Passage details/history ^a^ | NA  AAS ^b^ | NA GISAID  Acc. No. | Country |
| --- | --- | --- | --- | --- | --- | --- | --- |
| 1 | A/Khabarovsk/RII56/2016 | WHO National Influenza Centre Russian Federation | A(H1N1)pdm09 | C1 | S247R ^c^ | EPI835957 | Russian Federation |
| 2 | A/Aichi/83/2016 | National Institute of Infectious Diseases (NIID) | A(H1N1)pdm09 | Original | H275Y/H | EPI827140 | Japan |
| 3 | A/Egypt/1239/2016 | Crick Worldwide Influenza Centre | A(H1N1)pdm09 | cs | H275Y/H | EPI824250 | Egypt |
| 4 | A/England/60880155/2016 | Microbiology Services Colindale, Public Health England | A(H1N1)pdm09 | Original | H275Y/H | EPI878600 | United Kingdom |
| 5 | A/England/61660064/2016 | Microbiology Services Colindale, Public Health England | A(H1N1)pdm09 | Original | H275Y/H, S247S/N | EPI878091 | United Kingdom |
| 6 | A/Norway/1759-3/2016 | Norwegian Institute of Public Health | A(H1N1)pdm09 | Clinical Specimen | H275Y/H | EPI759192 | Norway |
| 7 | A/Vaxjo/1/2016 | Swedish Institute for Infectious Disease Control | A(H1N1)pdm09 | Direct | H275Y/H | EPI753099 | Russian Federation |
| 8 | A/Athens.Gr/729/2016 | Hellenic Pasteur Institute | A(H1N1)pdm09 | C1 | H275Y | EPI711180 | Greece |
| 9 | A/Belgium/H0008/2016 | Scientific Institute of Public Health (WIV-ISP) | A(H1N1)pdm09 | unknown | H275Y | EPI718572 | Belgium |
| 10 | A/Brazil/0257 S2/2016 | Centers for Disease Control and Prevention | A(H1N1)pdm09 | Original | H275Y/H | EPI827683 | Brazil |
| 11 | A/Czech Republic/14/2016 | Crick Worldwide Influenza Centre | A(H1N1)pdm09 | C3 | H275Y | EPI774689 | Czechia |
| 12 | A/Dijon/699/2016 | Institut Pasteur | A(H1N1)pdm09 | C2 | H275Y | EPI746263 | France |
| 13 | A/Egypt/1424/2016 | Crick Worldwide Influenza Centre | A(H1N1)pdm09 | cs | H275Y | EPI824334 | Egypt |
| 14 | A/Gunma/164/2016 | National Institute of Infectious Diseases (NIID) | A(H1N1)pdm09 | Original | H275Y | EPI855432 | Japan |
| 15 | A/Karlstad/3/2016 | Swedish Institute for Infectious Disease Control | A(H1N1)pdm09 | Direct | H275Y | EPI710059 | Russian Federation |
| 16 | A/Kursk/crie-305/2016 | Central Research Institute for Epidemiology | A(H1N1)pdm09 | MDCK | H275Y | EPI827054 | Russian Federation |
| 17 | A/Kursk/crie-306/2016 | Central Research Institute for Epidemiology | A(H1N1)pdm09 | MDCK | H275Y | EPI827056 | Russian Federation |
| 18 | A/Madrid/1860/2016 | Instituto de Salud Carlos III | A(H1N1)pdm09 | Clinical Specimen | H275Y | EPI758692 | Spain |
| 19 | A/Massachusetts/06/2016 | Centers for Disease Control and Prevention | A(H1N1)pdm09 | Original | H275Y | EPI756732 | USA |
| 20 | A/Murcia/2010/2016 | Instituto de Salud Carlos III | A(H1N1)pdm09 | Clinical Specimen | H275Y | EPI760887 | Spain |
| 21 | A/Nagasaki/15N002/2016 | Niigata University | A(H1N1)pdm09 | MDCK2 | H275Y | EPI704040 | Japan |
| 22 | A/Nagasaki/15N005/2016 | Niigata University | A(H1N1)pdm09 | MDCK2 | H275Y | EPI704047 | Japan |
| 23 | A/Niigata/15F255/2016 | Niigata University | A(H1N1)pdm09 | MDCK2 | H275Y | EPI712184 | Japan |
| 24 | A/Niigata/15F341/2016 | Niigata University | A(H1N1)pdm09 | MDCK2 | H275Y | EPI712577 | Japan |
| 25 | A/Niigata/15NU001/2016 | Niigata University | A(H1N1)pdm09 | MDCK2 | H275Y | EPI704049 | Japan |
| 26 | A/Norway/1476/2016 | Norwegian Institute of Public Health | A(H1N1)pdm09 | Clinical Specimen | H275Y | EPI759188 | Norway |
| 27 | A/Norway/1759-2/2016 | Norwegian Institute of Public Health | A(H1N1)pdm09 | Clinical Specimen | H275Y | EPI759191 | Norway |
| 28 | A/Norway/1828/2016 | Norwegian Institute of Public Health | A(H1N1)pdm09 | Clinical Specimen | H275Y | EPI759193 | Norway |
| 29 | A/Norway /2114/2016 | Norwegian Institute of Public Health | A(H1N1)pdm09 | Clinical Specimen | H275Y | EPI759195 | Norway |
| 30 | A/Norway/2404/2016 | Norwegian Institute of Public Health | A(H1N1)pdm09 | Clinical Specimen | H275Y | EPI759197 | Norway |
| 31 | A/Norway/411/2016 | Norwegian Institute of Public Health | A(H1N1)pdm09 | Clinical Specimen | H275Y | EPI759182 | Norway |
| 32 | A/Norway/541/2016 | Norwegian Institute of Public Health | A(H1N1)pdm09 | Clinical Specimen | H275Y | EPI759183 | Norway |
| 33 | A/Saint-Petersburg/ RII136/2016 | WHO National Influenza Centre Russian Federation | A(H1N1)pdm09 | CX | H275Y | EPI836133 | Russian Federation |
| 34 | A/Saint-Petersburg/RII150/2016 | WHO National Influenza Centre Russian Federation | A(H1N1)pdm09 | C1 | H275Y | EPI836398 | Russian Federation |
| 35 | A/Scotland/60180122/2015 | Microbiology Services Colindale, Public Health England | A(H1N1)pdm09 | Original | H275Y | EPI712067 | United Kingdom |
| 36 | A/Scotland/60240321/2016 | Microbiology Services Colindale, Public Health England | A(H1N1)pdm09 | Original | H275Y | EPI712120 | United Kingdom |
| 37 | A/Singapore/KK0381/2016 | Ministry of Health, Singapore | A(H1N1)pdm09 | Clinical Specimen | H275Y | EPI874185 | Singapore |
| 38 | A/St. Petersburg/136/2016 | Centers for Disease Control and Prevention | A(H1N1)pdm09 | C1 | H275Y | EPI828039 | Russian Federation |
| 39 | A/Toyama/86/2016 | National Institute of Infectious Diseases (NIID) | A(H1N1)pdm09 | Original | H275Y | EPI841478 | Japan |
| 40 | A/Vologda/CRIE-234/2016 | Central Research Institute for Epidemiology | A(H1N1)pdm09 | Original | H275Y | EPI826972 | Russian Federation |
| 41 | A/Michigan/58/2016 | Centers for Disease Control and Prevention | A(H1N1)pdm09 | Original | H275Y | EPI772226 | USA |
|  |  |  |  |  |  |  |  |
| 1 | A/England/917/2016 | Microbiology Services Colindale, Public Health England | A(H3N2) | Original | E119V | EPI875305 | United Kingdom |
|  |  |  |  |  |  |  |  |
| 1 | B/Netherlands/2518/2016 | National Institute for Public Health and the Environment (RIVM) | B Victoria | MDCK-I-P4 | E105K | EPI868728 | Netherlands |
| 2 | B/Bretagne/399/2016 | Institut Pasteur | B Victoria | C1 | D197N | EPI733495 | France |
| 3 | B/Singapore/INFKK-16-0467/2016 | Ministry of Health, Singapore | B Victoria | Clinical Specimen | D197N | EPI784541 | Singapore |
| 4 | B/Nordrhein-Westfalen/81/2016 | Robert-Koch-Institute | B Victoria | Original | I221V | EPI861193 | Germany |
| 5 | B/Novosibirsk/115/2016 | Centers for Disease Control and Prevention | B Victoria | C1 | K360E | EPI826581 | Russian Federation |
| 6 | B/England/959/2016 | Microbiology Services Colindale, Public Health England | B Victoria | Original | K360E | EPI877979 | United Kingdom |
| 7 | B/Champagne_Ardenne/1899/2016 | Institut Pasteur | B Victoria | unknown | D432G | EPI772685 | France |
| 8 | B/Laos/1471/2016 | Centers for Disease Control and Prevention | B Victoria | C1 | N294S | EPI854390 | Lao People’s Democratic Republic |
|  |  |  |  |  |  |  |  |
| 1 | B/Singapore/TT393/2015 | Ministry of Health, Singapore | B Yamagata | Clinical Specimen | D197N | EPI717987 | Singapore |
| 2 | B/Singapore/GP1165/2015 | Ministry of Health, Singapore | B Yamagata | MDCK | N294S | EPI718013 | Singapore |

^a^ Passage as shown in the sequence databases.

^b^ NA numbering is subtype-specific. NA amino acid substitutions (AAS) associated with reduced inhibition, as listed in the summary table provided by the AVWG on the WHO website (<http://www.who.int/influenza/gisrs_laboratory/antiviral_susceptibility/avwg2014_nai_substitution_table.pdf> ).

^c^ Described in reference Mandal et al., 2017.
